# Pharmacokinetics and Pharmacodynamics of Gliclazide from Immediate and Modified Release Formulation Tablets in Rats

**Published:** 2014

**Authors:** M Resztak, TW Hermann, W Sawicki, DZ Danielak

**Affiliations:** a *Department of Physical Pharmacy and Pharmacokinetics, K. Marcinkowski University of Medical Sciences, Poland*; b *Department of Pharmaceutical Technology, Medical University of Gdańsk, Poland*

**Keywords:** Gliclazide, Immediate release, Modified release, Pharmacokinetics, *In-vitro*, *in-vivo* correlation, HPLC

## Abstract

The objective of the study was to compare pharmacokinetic and pharmacodynamic parameters of gliclazide after administration of immediate (IR) and modified release (MR) tablets. The experiment included rats with both normoglycemia and streptozocin (STZ)-induced hyperglycemia. Several MR formulations were designed and *in-vitro* drug release profile was assessed by a dissolution test. For the further in-vivo study the most suitable formulation was chosen. For pharmacokinetic analysis concentrations of gliclazide in plasma were determined by a validated high performance liquid chromatography (HPLC) method with UV detection. Pharmacodynamic efficacy of the drug was evaluated by measuring blood glucose concentrations. Gliclazide bioavailability was totally different for two formulations in both healthy and diabetic rats based on area under the curve (AUC), time to peak concentration (tmax) and peak concentration (Cmax). Reduction of blood glucose level was significantly higher after the administration of IR than MR formulation. The highest pharmacodynamic efficacy of gliclazide was observed in the normal animals group after administration of the IR tablets, while hypoglycemic effect of the drug was diminished in animals with induced diabetes. Our study suggested that results of reduction in blood glucose level for STZ-induced groups were not comparable with pharmacodynamic effect for normal group. It may be assumed that a decrease in glycemia in healthy subjects might not be a suitable factor for characterizing anti-diabetic drugs.

## Introduction

Diabetes mellitus is a major health problem and an important cause of prolonged ill health and early death (1). It is a chronic metabolic disorder characterized by elevated blood glucose level and disturbances in carbohydrate, fat and protein metabolism. An uncontrolled diabetes leads to several complications, which include cardiovascular, renal, retinal and fungal infection Type 2 diabetes is the most common form of diabetes mellitus, accounting for approximately 90% of cases and affecting about 100 million people in the word. Projection indicates that there will be over 450 million type 2 diabetic patients by the year 2030 (2, 3). 

Diabetes mellitus is a metabolic disorder in which prolonged treatment is necessary. Maintenance of normal blood glucose level is essential in this condition, since both hyperglycemias as well as hypoglycemia are unwanted effects. An ideal anti-diabetic drug would be the one that not only does control the glycemia level but also prevents the development of complications (4). Oral hypoglycemic agents like sulphonylureas are still the major players in the management of type 2 diabetes. Gliclazide, a second generation sulphonylurea, is preferred in the therapy because of its selective inhibitory activity towards pancreatic K^+^ ATP channels, unique antioxidant properties and other beneficial haemobiological effects. Conventional formulations in long-term therapy for the treatment of chronic disease conditions require multiple dosing and therefore are disadvantageous. Therefore modified release (MR) tablets are preferred for such therapy because they allow better patient compliance, maintenance of uniform drug levels, decreased the fluctuation of peak-trough concentration and reduction of side effects (5, 6). 

We have reported previously on the MR tablets formulations of gliclazide (7). It has also been reported on the bioavailability of several commercial gliclazide products as classic IR tablets (including Diabrezide from Molteni and Diabezidum from Jelfa) and MR tablets (including Diaprel MR 30 mg and Diaprel 80 mg from Servier) available on the market. *In-vivo* C_max_ values were relatively high for IR gliclazide (4.12 and 3.44 mg/L^-1^, respectively) (8), as compared to the gliclazide MR formulations mentioned above (0.74 and 0.66 mg/L^-1^, respectively) (9, 10). Study on the pharmacotherapy of Type 2 diabetes shows that neither IR nor MR gliclazide tablet is capable of providing the therapeutic levels of gliclazide.

The following study was designed to evaluate pharmacokinetics and efficacy of glucose-lowering effect of gliclazide IR and MR tablets in normoglycemic and streptozocine induced hyperglycemia rats. The relationship of pharmacokinetics and pharmacodynamics of gliclazide in diabetic subjects was widely discussed in previously published papers (11, 12). In study of Stetinova *et al*., in which animal model was employed, it was acknowledged that alloxan-induced diabetes may influence pharmacodynamic response, as well as pharmacokinetics of gliclazide. However in that experiment the drug was administered as a suspension, while the most popular formulation is tablet. Therefore in our study we examined whether diabetes has an influence on pharmacokinetics and pharmacodynamics of gliclazide after single administration of either IR and MR minitablet.

## Experimental

*Drug and reagents*

Gliclazide and glibenclamide were purchased from Sigma-Aldrich Chemie, Germany. The potassium dihydrogen phosphate, sodium hydroxides were of reagent grade, and purchased from POCH, Poland. Methanol, acetonitryle were of HPLC grade and obtained from Merck, Germany, streptozotocine from Sigma Chemical, USA. Deionized water was always used (USF, Germany). Diabrezide tablets 80 mg lot no. 0074103 were purchased from Molteni, Italy. The following excipients were used for matrix tablet formulations: Kollidon SR (lot no. 85-3597, BASF, Germany); lactose FP V (Pharma Cosmetic, Poland); maltodextrin MD 200 (Grain Processing Corporation, USA); magnesium stearate (SO.G.I.S. Chimica Ind, Italy); talk (Farm-Impex, Poland). All the other chemicals used were of the analytical grade. 

*Apparatus and conditions*

A Specol UV VIS device (Carl Zeiss, Germany) was used for quantitative analyses of gliclazide the dissolution test. As a pH-meter we used a Cyberscan 500 pH (Eutech Instruments, Singapore). The dissolution of gliclazide from tablet formulations was evaluated in a Dissolution Tester type DT 60 paddle apparatus (Erweka, Germany). A Korsch EK-O/DMS laboratory press provided instrumentation to produce matrix formulation tablets. A HPLC system (Spectra Physics, USA) consisted of a pump and a variable wavelength detector.

*Formulations*

Several formulation tablets of gliclazide MR (G - 1 – G - 4) were developed. The IR formulation (D - 1) consisted of pulverized, commercially available tablet (Diabrezide). The compositions of fabricated formulations with their codes are shown in Table 1. The 3 mg amounts of gliclazide were kept constant for all the formulations and the amount of Kollidon SR decreased gradually for each set of formulation. 

Tablets discs (3 mm in diameter) of the formulations were compressed at 12 kN using Shimadzu press. The weight of each tablet was determined ( 23.18 ± 1.2 mg and 25.14 ± 0.6 mg mean weight of MR and IR tablets, respectively). 

**Table 1:** Ingredients (%) of the fabricated gliclazide matrix tablets (MR).

**Ingredients (%)**	**Formulations**			
	**G-1**	**G-2**	**G-3**	**G-4**
**Gliclazide**	13.4	13.4	13.4	13.4
**Lactose**	33.5	15.6	24.6	29.0
**Maltodextrin**	6.7	6.7	6.7	6.7
**Kollidon SR**	44.7	62.6	53.6	49.2
**Sodium stearic fumarate**	1.7	1.7	1.7	1.7

*In-vitro study*

*In-vitro* dissolution rates of gliclazide from matrix formulations were obtained (on 6 tablets of each formulation) using rotating paddles at 100 revolutions per minutes according to the specifications of the BP 2008 (apparatus II). The dissolution medium was 500 mL of phosphate buffer at pH 7.4 maintained at 37 °C throughout the experiment. Aliquots of 5 mL were collected at regular intervals up to 8 h after the commencement of the experiment. Gliclazide concentrations were determined with a UV spectroscopic method ( = 226 nm) as described previously (7).

Different mathematical models (zero-order, first-order and Higuchi (13)) for simulation of kinetics of the drug-release process from matrix tablets were applied and the best fitting model was chosen. 

Furthermore, for better characterization of the drug release profile the Korsmayer-Peppas (Eq. 1) model was utilized (14):

(1) $$M_{t}/M_{\infty }={\mathrm{kt}}^{n}$$

Where M_t_ and M_∞ _are cumulative amounts of drug released at time t and at infinite time respectively, k is a constant comprising the structural and geometric characteristics of the tablet, and n (the release exponent) is a parameter which depends on the release mechanism and is thus used to characterize it.

The mean dissolution time (MDT) was calculated from dissolution data, according to Mockel and Lippold using the following equation (15):

(2) $$\mathrm{MDT}=\left(\frac{n}{n}+1\right).k^{-1/n}$$

For further *in-vivo* studies the MR formulation that provided the slowest *in-vitro* dissolution rate was selected.

*In-vivo study*

*Animals*

Ten week - old Wistar rats both sexes weighing between 255 - 284 g were used in the study. They were maintained a standard palled diet and water. The animals were fasted for 12 h before experiment and food was withdrawn during the experiment.

The animal experiments conducted were approved by Institutional Animals Ethics Committee at the University of Medical Sciences in Poznań (No 24/2006) and adhere to the Principles of Laboratory Animal Care.

*Induction of diabetes*

Neonatal rats (5 days old) were used for inducing type 2 diabetes. A dose of streptozotocin equivalent to 80 mg/Kg body weight was dissolved in a sodium citrate buffer (pH 4.5) and administered intraperitoneally to the five days old animals. The dose of streptozocin used in the study was slightly modified from the method of Adikwu *et al*. (16), who used a lower dose of the drug (60 mg/Kg) to induce diabetes. Control group (non-diabetic rats) received citrate buffer alone, 10 weeks after STZ injection, oral glucose tolerance test was performed to evaluate the extent of diabetes induction. Glycemia was measured in fasting conditions and 1 hour after administration of glucose (1 g of anhydrous glucose/Kg). Glucose concentrations were measured from a blood drop using a glucometer-strip system (AccuCheck Active glucometer). Rats were considered type 2 diabetic and included in the study when glucose level after administration of glucose was 150-350 mg/dL. 

*Dosing and sampling*

Both STZ-induced diabetic and non-diabetic rats were divided into three equinumerous (n = 7) subgroups. Each subgroup receives either a placebo (methylcellulose solution), a gliclazide IR or a gliclazide MR formulation. Non-diabetic rats were used as a control group. Minitablets of gliclazide were directly injected into the stomach by intragastric gavage. Minitablets were firmly placed in the flat point end of the needle which allowed safe administration of chosen formulation. Blood samples (300 µL) were withdrawn from the tail vein of each rat before and at 1, 2, 4, 6, 8 hours after administration of the IR tablets or placebo and at 2, 4, 6, 8 and 12 hours after MR tablets administration. Collected samples were immediately centrifuged at 5000 rpm for 15 min and separated plasma was frozen at -20 ˚C for further analysis of gliclazide. Simultaneously, glucose levels were measured as described above.

*Gliclazide plasma assay*

A simple and rapid method was used to analyse gliclazide in serum by the HPLC method that was the modification of the previously reported procedure by Główka *et al*. (17). In the modified method only 80 µL of plasma is required for the analysis.

Gliclazide and glibenclamide (I.S.) were extracted from rat's plasma by solid phase extraction (SPE). Baker Bond C 18 colums (J.T. Baker, Netherlands) were preconditioned with 2 x 1 mL methanol and 2 x 1 mL distilled water. Subsequently samples were eluted with 3 x 100 µL methanol and evaporated under gentle nitrogen stream at 40 ^0^C. LiChrospher 100 C 18 (5 µm) 250 x 4 mm with a precolumn LiChroCart 4-4 packed with a LiChrospher C 18 (5 µm) sorbent (both Merck, Germany) were used as analytical columns. The mobile phase was a mixture of 0.04 M potassium dihydrogenphosphate (pH = 3.8) and acetonitrile (51: 49; v/v). Flow rate was 1 mL/min and the gliclazide peaks were detected at 226 nm.

Gliclazide calibration curves were linear in the range 0.2 - 18 µg/mL and 20 - 50 µg/mL with a correlation coefficient of 0.999. The limit of quantification (LOQ) and the limit of detection (LOD; signal to noise ratio of 3) for gliclazide in serum were 0.1 µg/mL and 0.05 µg/mL, respectively. 

*Pharmacokinetic, pharmacodynamic and statistical analysis*

Pharmacokinetic parameter estimates were calculated using TOPFIT 2.0 software, based on the non - compartmental model. Statistical analysis was performed using STATISTICA 7.0 (StatSoft Inc., USA) software. The results were expressed as a mean ± standard deviation (SD). Student t-test was used for comparison of the differences between formulations. A value of p < 0.05 was considered statistically significant. 

*In-vitro-in vivo correlation (IVIVC)*

The main purpose of an IVIVC model is to utilize *in-vitro* dissolution profiles as a surrogate for *in-vivo* bioequivalence and to support biowaivers and data analysis of IVIVC attracts attention from the pharmaceutical industry and also to predict the entire *in-vitro* time course from the *in-vitro* data. There are total five levels of correlation *i.e*. A, B, C, D and multiple Level C (18, 19).

In this work level A of correlation was studied. This level of correlation is the highest category of correlation an represents a poin-to-point relationship between *in-vitro* dissolution rate and *in-vivo* input rate of the drug from the dosage form. The first step was to calculate the fraction of the drug absorbed. The Wagner-Nelson method and a module of TOPFIT 2.0 software were utilized for this calculation. The second step was comparison of the fraction of drug absorbed to the fraction of drug dissolved in order to construct a level A IVIVC. The linear regression analysis was used for examination the relationship between percent of drug dissolved and percent of drug absorbed. The following equation was used (19):

% absorbed in-vivo(t)=% dissolved in-vitro(t)∙FabsMR(t)          (3)

were % *in-vitro* dissolved (t) is the *in-vitro* dissolution at time t, % *in-vivo* input (t) is the percent of dose introduced to the systemic circulation at time t and Fabs_MR_ (t) is the slope of the regression line and stands for the fraction of dose absorbed from MR formulations at time t. 

## Results

**Figure 1:**
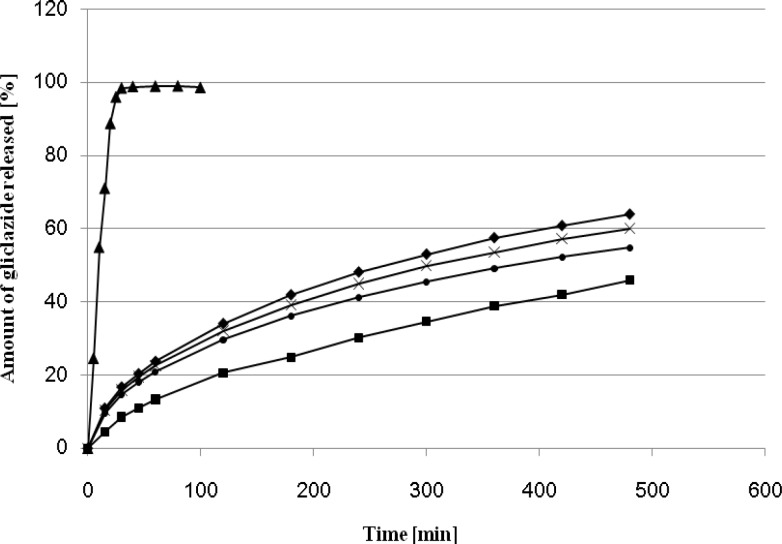
Release profiles of gliclazide from MR formulations (G-1 - G-4) and IR formulation (D-1).

The effect of different amount of Kollidon SR on gliclazide release rate is presented on Figure 1. 

The use of Kollidon SR as a plastic material in the direct compression process to formulate a sustained release dosage form has been reported previously (7, 20). It is particularly suitable for the manufacture of pH-independent sustained release matrix tablets. A significant effect of Kollidon on the release profile was observed when its amount exceeded 45 % and 49 %. On 8 hours elapsed 64 % and 60 % of drug were released from the matrix formulations G - 1, G - 4. The optimum release profile (45.88 % after 8 hours) was observed for the formulation containing 63 % of Kollidon SR, The dissolution profile of the formulation G - 2 was the most similar to that observed for Diaprel and formulation C described in our previous paper (7). On this basis the tablets G - 2 were selected for further *in-vivo* studies in rats.

The values of release exponent (n), kinetic rate constant (k) and mean dissolution time (MDT) for all formulations were calculated from Equation 1 and Equation 2, and are presented in Table 2. As observed from obtained results, the value of correlation coefficient (r^2^) for all formulations were high enough to evaluate the drug dissolution behavior by Equation 1.

**Table 2:** The values of the release exponent (n), kinetic constant (k), correlation coefficient (r2), and mean dissolution time (MDT) for MR (G) and IR (D) gliclazide formulation tablets.

**Formulation Tablets**	**n**	**k**	**r2**	**MDT (min)**
G-1	0.45	0.0662	0.9985	128.7
G-2	0.45	0.070	0.9976	113.7
G-3	0.45	0.0654	0.9985	132.2
G-4	0.45	0.0658	0.9988	130.4
D-1	0.45	0.2982	0.9703	4.6

**Figure 2:**
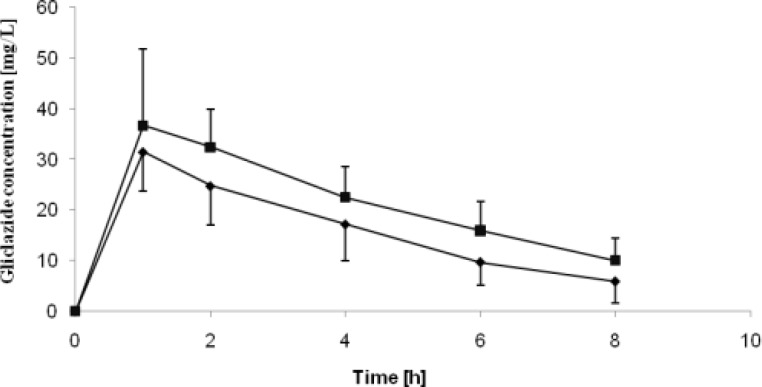
Mean gliclazide pharmacokinetic curves obtained on healthy and STZ-treated rats treated with IR formulation of gliclazide

**Figure 3:**
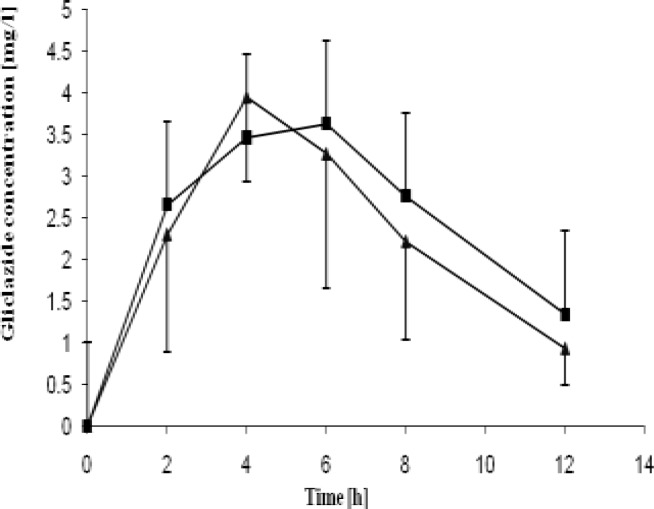
Mean gliclazide pharmacokinetic curves obtained on healthy and STZ-treated rats with treated MR formulation of gliclazide

The pharmacokinetic parameters of gliclazide acquired in the study are presented in Table 3. Gliclazide plasma concentration curves are presented in Figure 2 and Figure 3. Obtained C_max_ values were over 8-fold greater than MR tablets for both healthy and diabetic rats. The IR tablets were also characterized by shorter T_max_ for both groups as compared to MR formulations. The significantly higher AUC values observed for conventional tablets indicate increased bioavailability of the drug from IR formulations if compared to the MR formulations. Bioavailability of IR tablets was 6-fold greater if compared to MR tablets . 

**Table 3:** Mean pharmacokinetic parameters (± SD) of gliclazide after oral administration of the IR and MR formulation minitablets in rats.

Parameter (units)	IR formulation			MR formulation		
	Diabetic rats	Normal rats	p	Diabetic rats	Normal rats	p
C_max_ (µg/mL)	38.84±11.48	31.60±9.11	0.225	4.20±1.33	4.53±2.22	0.908
t_1/2_ (h)	3.47±1.28	2.58±1.08	0.142	3.84±1.54	3.53±1.58	0.418
k_e _(1/h)	0.22±0.08	0.31±0.11	0.142	0.20±0.06	0.26±0.14	0.418
AUC_0→∞ _(µg·h/mL)	243.90±110.37	155.57±76.14	0.110	32.87±10.32	34.37±6.03	0.203
MRT (h)	5.84±1.87	4.40±1.37	0.064	7.81±1.57	6.93±0.89	0.132
**t**_max _ **(h)**	0.24±0,11	0.40±0.20	0.110	1.65±0.49	2.08±0.69	0.203

Statistical tests were performed to evaluate differences between mean values of glucose blood concentrations after administering either a placebo or a certain gliclazide formulation tablet. In IR gliclazide groups the hypoglycemic effect was statistically different (p < 0.05) 2 - 8 hours after the tablet was taken for both diabetic and normal rats. Nevertheless lowering of glycemia was greater in normal than in diabetic rats: after 1 hour glucose concentration dropped by 47 % in normoglycemic animals while in the STZ group it was reduced by 25 % only (Table 4).

**Table 4:** Effect (%) of gliclazide IR formulation minitablets on the plasma glucose levela in normal and STZ- treated rats.

**Group**	**Time (h)**						
	**0** **b**	**1**	**2**	**4**	**6**		**8**
Normal rats (Control group)	107.63±4.34 (100%)	129.63±9.24 (120.79±12.13%)	132.50±8.02 (123.38±10.42%)	127.38±8.31 (118.63±10.67%)	126.13±7.28 (117.41±9.15%)	123.63±2.50 (115.04±5.33%)	
MR formulation	102.29±5.25 (100%)	99.29±11.10 (97.06±9.41%)	103.71±11.07 (101.67±12.26%)	111.43±15.79 (109.33±17.23%)	120.14±11.36 (117.78±13.01%)	119.00±6.24^ c^ (116.52±6.84%)	
STZ-treated rats (Control group)	115.43±8.92 (100%)	131.71±19.27 (114.59±17.78%)	140.71±19.81 (122.9±16.96%)	157.00±28.34 (137.01±26.92%)	150.29±30.32 (130.24±24.33%)	135.00±13.50 ^c^ (117.20±10.50%)	
MR formulation	103.14±14.54 (100%)	154.86±41.66 (154.38±51.81%)	127.00±16.07 (125.10±21,85%)	122.00±7.10 (120.15±16.66%)	127.43±8.38 (125.68±19.56%)	144.29±13.01 (142.23±22.23%)	

There was a discrepancy in MR treated animals. In normal rats a significantly lower glucose level was maintained in 2, 4, and 6 hour while in the diabetic rats group such difference occurred only at 6 and 8 hour after the administration of gliclazide (Table 5).

**Table 5:** Effect (%) of gliclazide MR minitablets on the plasma glucose level^a^ in normal and STZ- treated rats

**Group**	**Time (h)**							
	**0** ^b^	**1**	**2**	**4**		**6**		**8**
Normal rats (Control group)	107.63±4.34 (100%)	-	129.63±9.24 (120.79±12.13%)		132.50±8.02 (123.38±10.42%)	127.38±8.31 (118.63±10.67%)	126.13±7.28 (117.41±9.15%)	
IR formulation	112.43±7.25 (100%)	58.86±3.98 (52.56±5,35%)	59.86±14.71 (53.02±11.08%)		87.29±13.16 (77.49±9.28%)	90.00±18.79 (79.69±13.40%)	90.57±21.69 (80.18±16.31%)	
STZ-treated rats (Control group)	115.43±8.92 (100%)	-	131,.1±19.27 (114.59±17.78%)		140.71±19.81 (122.19±16.96%)	157.00±28.34 (137.01±26.92%)	150.29±30.32 (130.24±24.33%)	
IR formulation	114.57±5.74 (100%)	83.1±16.96 (72.86±12.69%)	87.14±10.02 (76.07±7.79%)		95.00±6.88 (82.86±2.91%)	101.00±12.11 (87.97±7.24%)	109.00±19.79 (94.72±13.35%)	

^a^ Plasma glucose levels are expressed as mg/dL

^b^ Initial values of glucose blood concentration before gliclazide administration are considered as to 100%

the mean ± standard deviation (n = 7)

^c ^Statistical significance at p < 0.05 (compared to the control group)

The *in-vitro, in-vivo* correlation (IVIVC) was determined by plotting a graph of the fraction of absorbed drug *in-vivo* versus the fraction of drug released *in-vitro*. A high value of correlation coefficient (r^2 ^= 0.97) indicated good correlation between *in-vitro, in-vivo* data.

## Discussion

Although the Fickan mechanism is typical for IR gliclazide formulation tablets, the non - Fickan mechanism characterizes the release of gliclazide from its matrix formulation tablets (20). Then value demonstrates that the transport mechanism of gliclazide from all formulation tablets is a Fickan one (n = 0.45) (Table 2). Comparison of MDT showed that the G - 3 formulation produced the best release-sustaining properties. On the other hand the formulation G - 2 presented the most similar release profile to MR tablets available on the market (Diaprel MR). On this basis the G - 2 as MR formulation was selected for further *in-vivo* studies in rats.

In the present study n5-STZ (streptozocin injection on the 5^th^ day of birth) rat model of diabetes was used. Depending on the day of streptozocin injection, various extent of β cell damage could be obtained. Type 2 diabetes mellitus is generated by injecting STZ intravenously or intraperitonelly (100 mg/Kg) on the day of birth (n0 - model) with of. The rats treated with STZ on the day of birth, exhibit insulin deficient acute diabetes mellitus 3 - 5 days after birth. However the hyperglycemia observed in the neonates following STZ is only transient and the plasma glucose and insulin values are no longer significantly different from those of control. It was found n0 - STZ rats showed mild hyperglycemia that only 8 weeks after STZ injection (21). Alternatively the n - STZ rat model was developed by varying the day of the STZ injection after the birth, such as 2^nd^ day or 5^th^ day of the birth. These are alternatively called n2 - STZ and n5 - STZ model respectively. The n2 - STZ and n5 - STZ models are developed by 80 mg/Kg *i.p*. STZ injection. The n0 - STZ and n2 - STZ models are found almost similar with respect to growth, basal plasma glucose, insulin levels, lack of insulin release in response to glucose, *in-vivo* glucose intolerance and depletion of pancreatic insulin stores. The n5 - STZ model shows an unaltered basal hyperglycemia, glucose intolerance, raised glycosylated hemoglobin, a strong reduction of the pancreatic insulin stores, decreased basal insulin levels and lack of plasma insulin response to glucose *in-vivo*. It is acknowledged that none animal model is identical to any human syndrome; none of the available animal models of type 2 diabetes mellitus exactly simulates the human type 2 diabetes mellitus. However, the development and progression of hyperglycamia found in the n5 - STZ Wistar models demonstrated many similarities and are considered to be one of the most suitable experimental animal models of type 2 diabetes mellitus (22).

The relationship between pharmacokinetics of IR and MR gliclazide formulations and their anti-diabetic efficacy was evaluated on the basis of experimental determination of changes with time in the plasma levels of this anti-diabetic agent and those of glucose in normal and diabetic rats. There was no-statistical difference (p > 0.05) of evaluated pharmacokinetic parameters between diabetic and control group in both IR and MR formulations, except for AUMC_0→∞_. In addition, no-significant differences were observed in t_1/2_, k_e_ and MRT between two formulations (p > 0.05) whereas other pharmacokinetics parameters of IR tablets were significantly different from those of MR in normal and diabetic rats (p < 0.05). The low elimination rate constants (k_e_) and high mean residence time (MRT) confirm the sustained release mechanism of the MR drug formulations In addition the investigated MR tablets were capable of maintaining constant plasma gliclazide concentration throughout the time of experiment. Therefore necessity of frequent administration is eliminated as well as occurrence the dose-dependent side effects associated with administration of conventional gliclazide tablets. T_1/2_ and k_e_ were similar for IR and MR formulation, although mean values suggest slower elimination of gliclazide in MR tablet group. Surprisingly, we observed a discrepancy in pharmacokinetics of gliclazide between diabetic and healthy rats. Even though statistical test showed no-significant difference between those two groups, a tendency towards slower elimination in diabetic rats was observed. Moreover, bioavailability of gliclazide as well as C_max_ was higher in STZ-group, but only for IR tablet. 

Pharmacodynamics of formulations was examined through analysis of glucose level changes in time. Obtained values were analyzed for both gliclazide and placebo administered groups. When pharmacodynamic effect is considered it seems that gliclazide was more effective in lowering blood glucose levels in normal rats than in diabetic ones. The most significant reduction in glucose concentration was observed in the normal rats group treated with gliclazide IR. One hour after administration it was lowered by almost 50 mg/dL if compared to the initial concentration. Sudden drop of glucose concentration may lead to many symptoms such as hunger, extensive sweating, behavioral changes and even epileptic events. In case of gliclazide MR tablets the glucose concentrations were maintained on a relatively stable level, however the hypoglycaemic effect seemed to be larger in normal rats, whereas in STZ-treated individuals it oscillated around concentrations measured for placebo. This difference might be an effect of decreased number of -cells or their impaired function after STZ injection. Stetinova* et al*. (11) observed similar results in rats with alloxan-induced diabetes.

The IVIVC is a predictive mathematical model describing the relationship between *in-vitro* property of a dosage and relevant *in-vivo* response (23). As the correlation was established the expected bioavailability of MR formulation can be accurately and precisely predicted from dissolution profile characteristics. Therefore, dissolution tests may be used as a sensitive, reliable and reproducible surrogate for bioequivalence tests. As were as, the technological parameters like equipment, manufacturing process, batch size may be optimized. The Food and Drug Administration (FDA) guidance has identified four categories of IVIVC models: namely, level A, B, C and multiple C models (19). Several investigators (23, 24 and 25) have attempted to develop IVIVC models based on these categories. Therefore, in certain cases, especially for MR formulations, the dissolution test can serve as an indicator of *in-vivo* performance of a formulation. IVIVC may reduce the number of bioequivalence studies that must be performed during initial approval process and also post-approval changes. Gliclazide properties such low water solubility and a high lipophilicity classify this drug to the second class of the BCS, for which the level A of IVIVC is expected. In this case, a formulation may influence the *in-vitro* dissolution rate leading to different absorption rates (18). 

In summary, bioavailability of MR tablets was 6-fold lower if compared to conventional tablets. The difference in IR gliclazide plasma concentration between normoglycemia and streptozocin-treated groups was greater if compared to MR gliclazide (the levels in STZ-treated animals tended to by slightly higher). The pharmacodynamic efficacy of gliclazide was less significant in the animals with streptozocin-induced hyperglicemia if compared to normoglycemic rats. It seems that a decrease in glycemia in healthy subjects might not be a suitable factor for characterizing antidiabetic drugs.
